# Relationship between Vitamin Intake and Depressive Symptoms in Elderly Japanese Individuals: Differences with Gender and Body Mass Index

**DOI:** 10.3390/nu9121319

**Published:** 2017-12-03

**Authors:** Thao Thi Thu Nguyen, Hiromasa Tsujiguchi, Yasuhiro Kambayashi, Akinori Hara, Sakae Miyagi, Yohei Yamada, Haruki Nakamura, Yukari Shimizu, Daisuke Hori, Fumihiko Suzuki, Koichiro Hayashi, Hiroyuki Nakamura

**Affiliations:** Department of Environmental and Preventive Medicine, Graduate School of Medical Science, Kanazawa University, 13-1 Takara-machi, Kanazawa 920-8640, Japan; toi_fs@yahoo.com (T.T.T.N.); t-hiromasa@med.kanazawa-u.ac.jp (H.T.); ykamba@med.kanazawa-u.ac.jp (Y.K.); ahara@m-kanazawa.jp (A.H.); smiyagi@staff.kanazawa-u.ac.jp (S.M.); yamada503597@gmail.com (Y.Y.); haruki_nakamura_kanazawa@yahoo.co.jp (H.N.); h_zu@me.com (Y.S.); hori_d@mbr.nifty.com (D.H.); fumi@dental.email.ne.jp (F.S.); orihciok1003@gmail.com (K.H.)

**Keywords:** depression, vitamin, elderly individuals, Japanese

## Abstract

Only a few studies have focused on the relationship between vitamin intake and depressive symptoms in Japanese individuals. This cross-sectional study investigated the relationship between vitamin intake and depressive symptoms in 1634 elderly Japanese individuals (65 years and older). The consumption of fifteen vitamins including retinol, a retinol equivalent, beta-carotene equivalent, vitamin D, alpha-tocopherol, vitamin K, vitamin group B, vitamin C, and cryptoxanthine was analyzed using a brief-type self-administered diet history questionnaire (BDHQ). The short version of the Geriatric Depression Scale (GDS) was used to assess depressive symptoms. The prevalence of participants with depressive symptoms was 26.7%. The consumption of all vitamins, except for retinol and vitamin D, was lower among depressed than non-depressed participants. The consumption of vitamins was significantly less in female and overweight participants with depressive symptoms than in elderly participants without depressive symptoms. After adjustments for potential confounders, none of the fifteen vitamins were correlated with depressive symptoms in male or underweight participants. Associations between vitamin deficiencies and depressive symptoms were observed in female and overweight elderly participants. Our findings demonstrated a relationship between vitamin intake and depressive symptoms.

## 1. Introduction

Depression is a major cause of illness in societies worldwide, affecting personal well-being, ability to work, and the use of healthcare resources [1]. The total estimated number of individuals living with depression globally is 322 million [2], with an increase of 18.4% being observed between 2005 and 2015 [3]. In elderly individuals, depression is considered to be the most frequent cause of emotional suffering and significantly decreases quality of life [4,5,6]. The prevalence of depression varies, and is higher among woman. Its prevalence in Korean middle-aged and older adults was previously reported to be 4.1% in men and 11.7% in women [7]. Another study on an older Chinese population suggested that the risk of depression was 1.98-fold higher in women than in men [8]. A study on 3113 community-dwelling individuals aged 40 years or older in Japan reported more severe depressive symptoms in 4.3% of male and 6.3% of female participants [9]. 

Previous studies have also investigated the reciprocal relationship between being overweight, obesity, and depression [10,11]. A cross-sectional study using Korean National Health Examination Survey data showed a relationship between a higher body weight and more severe depressive symptoms [12]. A three-year longitudinal epidemiological design among older adults suggested that body mass index (BMI) was associated with depressive symptoms; however, the effect size was small [13]. In contrast, other studies found a negative or no relationship between depression and being overweight/obese [14,15].

Depression in the elderly is complex and may occur for various reasons and under different conditions. Diet and nutrition, particularly vitamins, influence depression. Mikkelsen et al. have demonstrated in their studies that deficiency in vitamin B such as B1, B3, B6, B9, B12 has been linked to depression [16,17]. Previous findings indicated that fat-soluble antioxidant micronutrients, such as vitamin E, protect against neural damage, with a low dietary intake of vitamin E being related to altered mood and depression [18,19]. In other studies, a correlation was found between depression and decreased serum 25-hydroxyvitamin D (25(OH)D) levels in older individuals [20,21]. A systematic review and meta- analysis study have indicated an inverse association between serum 25(OH)D levels and the risk of depression [22]. However, another systematic review study has shown that the evidence linking vitamin D intake to non-clinically depressed in healthy individuals was inconsistent; larger, longer trials suggested that this evidence was not reliable [23]. Studies that have reviewed the association between vitamin A and depression have suggested that retinoic acid, an active form of vitamin A, can cause depression and suicide in some susceptible individuals [24,25]. The study by Oishi J showed that carotene, vitamin C intakes were associated with lowering depressive symptoms among community-dwelling elderly persons in Japan [26]. Intervention trials have also provided evidence for the effectiveness of vitamin supplements in the treatment of depression [27,28,29].

Although Japan was listed as a country with a lower prevalence of depression in a cross-sectional study on eighteen high- and low-to-middle-income countries [30], the number of patients with depression has been increasing [31]. Studies on vitamin intake have been conducted among elderly Japanese individuals; however, evidence of a relationship between vitamin intake and depression is limited, particularly in categorized groups. Therefore, we conducted this study on older individuals in order to investigate the relationship between vitamin deficiencies and depressive symptoms with differences in gender and BMI.

## 2. Materials and Methods 

### 2.1. Study Population 

The present cross-sectional study was based on the Shika Study, which has been conducted in Shika town, Ishikawa prefecture since 2011. The Shika Study included interviews, self-administered questionnaires, and a comprehensive health examination. 

**Figure 1:**
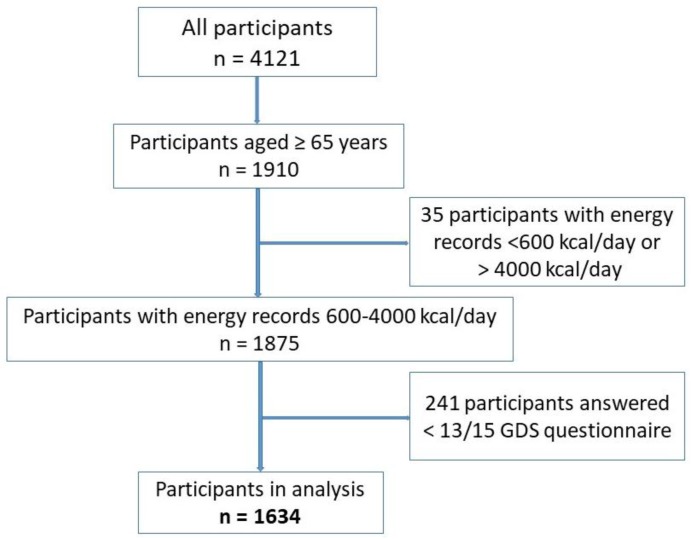
Participant recruitment chart.

Shika town is located in Ishikawa prefecture with a population of 21,061 individuals, of which approximately 40% are elderly (65 years and older) and 56% of individuals are older than 40 years of age. We selected four model districts in Shika town for the study including Horimatsu, Higashi-masuho, Tsuchida and Togi with a population in 2016 of 7183 and 2592 households [32]. The invitation letters to participate in the study were sent to all individuals from 40 years of age between January 2015 to January 2016. The study population of the Shika study consisted of 4121 individuals from 40 years of age. Persons were eligible to participate in the study if they were 65 or older and capable of giving informed consent. The present study was conducted on 1634 elderly participants aged 65 years and older (39.65% of all participants from 40 years of age). Written informed consent was obtained from all participants in the survey. The present study complied with Declaration of Helsinki Guidelines and it was approved by the Ethical Committee at Kanazawa University. The recruitment of subjects is shown in Figure 1.

### 2.2. Depression

Depressive symptoms were assessed using the short version of the Geriatric Depression Scale (GDS), a self-administered survey consisting of 15 yes/no questions with higher scores indicating higher depressive symptomatology [33]. A review article on the criterion validity of GDS has suggested that the most often a cut-off value of 5 or 6 was used [34]. A Japanese version of the 15-item GDS has been evaluated for validity and reliability in a Japanese population with the recommended optimal cut-off score of 6/7 [35]. In the present study, we used a predefined cut-off point of 6 to define depressive symptoms and non-depressive symptoms; subjects with a GDS score from 6 were categorized as having depressive symptoms , while those with a GDS score of less than 6 were categorized as having non-depressive symptoms (a cut-off score of 6 for the GDS had a sensitivity of 0.973 and specificity of 0.959) [36]. We only included participants who answered more than 13 out of the 15 questions in the analysis. 

### 2.3. Nutrients Assessment

A brief-type self-administered diet history questionnaire (BDHQ) was used to analyze nutritional intake including the consumption of 15 vitamins and their compounds: retinol, a retinol equivalent, beta-carotene equivalent, vitamin D, alpha-tocopherol, vitamin K, vitamin group B (B1, B2, B3, B5, B6, B9, and B12), vitamin C, and cryptoxanthine. In the present study, we used a validated BDHQ, which was designed to obtain information on each individual’s nutritional and food intakes as well as a few markers of dietary behavior during the previous month in order to assess the amount of nutrients habitually consumed from food typically eaten (excluding intake from dietary supplements) [37,38,39]. The BDHQ dietary intake was based on the consumption frequency of selected food and nonalcoholic beverage items, the daily intake of rice and miso soup, usual cooking methods for fish and meat, and general dietary behavior [38,39]. The estimated vitamin intake (g/day) for 58 food and beverage items, which are most commonly consumed in Japan with some modifications using a food list suggested by the National Health and Nutrition Survey of Japan [40], were calculated using an ad-hoc computer algorithm based on the Japanese standard of food composition table [41] which included weighting factors for the BDHQ [42].

We also adjusted the basic index from the BDHQ, including total energy, protein, lipid, and carbohydrate intakes. We excluded all participants who reported a total energy intake of less than 600 kcal/day (half of the required energy for the lowest physical activity category) or more than 4000 kcal/day (1.5 times the energy intake required for the moderate physical activity category) due to under/over-estimations leading to bias in the analysis of other nutrients [37,40,41].

### 2.4. Other Variables

BMI was calculated as current body weight (kg) divided by the square of body height (m). Subjects were grouped into 3 categories: underweight (BMI less than 18.5), normal weight (BMI ranging between 18.5 and 24.99), and overweight (BMI of 25 and higher). 

Participants also reported their living status (living alone or with someone), marital status (single, widowed, separated), smoking status (current smoker, had stopped, non-smoker), alcohol drinking, and history of chronic diseases (hypertension, stroke, myocardial infarction, diabetes, hyperlipidemia). In our population, participants who reported a history of stroke and/or myocardial infarction also had hypertension and/or diabetes. Therefore, we focused on hypertension, diabetes, and hyperlipidemia as confounders in the analysis.

### 2.5. Statistical Analysis

Vitamin intake was adjusted for energy using the density method as a percentage of the daily energy intake for energy-containing nutrients. Differences in characteristics and vitamin intake between participants with and without depressive symptoms were assessed using the χ^2^ test (categorical variables) and Student’s *t*-test (continuous variables).

Confounding variables were gender, age, height, weight, BMI, living conditions, marital status, smoking status, alcohol drinking, and a history of hypertension, diabetes, and hyperlipidemia. 

Participants were divided into gender and BMI groups for analyses. The odd ratios (ORs) and 95% confidence intervals (CIs) of each vitamin for depressive symptoms were calculated by univariate and multivariate logistic regression models with adjustments for potential confounding factors in order to assess the relationship between vitamin intake and depressive symptoms. Data were statistically analyzed using the SPSS software program for MS Windows, version 23.0 (SPSS, Inc., New York, NY, USA). The significance of differences was set at *p* < 0.05 for all analyses. 

## 3. Results

### 3.1. Participant Characteristics According to the Depressive Symptoms Status

Among 1634 participants (74.48 ± 7.43 years old), 55.94% (*n* = 914) were women and 437 (26.7%) exhibited depressive symptoms, of which 26.8% females (245/914) and 26.6% males (192/720) had depressive symptoms (Supplementary Table S1). Participants with depressive symptoms were significantly older than participants without depressive symptoms. In male participants, those with depressive symptoms had a significantly lower energy intake (1893.0 ± 582.1 kcal/day) than participants without depressive symptoms (2058.7 ± 644.4 kcal/day); however, they had a significantly higher carbohydrate intake (137.8 ± 21.8 compared to 133.9 ± 22.2 g/1000 kcal). Similar results were observed in females (Table 1).

Vitamin intake was compared between depressive symptoms and participants without depressive symptoms. The levels of thirteen out of fifteen vitamins were lower in participants with depressive symptoms, except for retinol and vitamin D. The levels of the entire vitamin group B were significantly lower in participants with depressive symptoms (Table 2).

### 3.2. Vitamin Intake in Different Groups of Participants 

In males, only vitamin K levels were significantly lower in participants with depressive symptoms (148.69 ± 83.04 mg/1000 kcal) than in participants without depressive symptoms (165.52 ± 89.82 mg/1000 kcal). In contrast, among females, the intake of all fifteen vitamins was significantly reduced in participants with depressive symptoms (Table 3).

By stratifying according to BMI, participants with an underweight BMI did not show any significant difference in vitamin intake between the group with depressive symptoms and the group without depressive symptoms. Similar differences were observed in normal BMI and overweight BMI participants. In normal BMI participants, the levels of nine out of fifteen vitamins and compounds were significantly lower in participants with depressive symptoms than in participants without depressive symptoms, including the beta-carotene equivalent, alpha-tocopherol, vitamin K, vitamin B1, vitamin B3, vitamin B6, vitamin B9, vitamin B5, and vitamin C. A number of differences were noted in fourteen vitamins and compounds (except for cryptoxanthine), such as a lower intake in overweight participants with depressive symptoms. The intake of the entire vitamin group B was higher in participants without depressive symptoms than in participants with depressive symptoms (Table 4).

### 3.3. Influence of Gender and BMI on the Relationship between Vitamin Intake and Depressive Symptoms

Vitamin K, vitamin C, vitamin B1, vitamin B5, vitamin B6, and vitamin B9 deficiencies correlated with depressive symptoms after adjustments for all covariates (Supplementary Table S2). We investigated whether a similar relationship existed between vitamin intake levels and depressive symptoms with differences in gender or BMI. No correlations were observed between vitamin intake and depressive symptoms in underweight participants (Supplementary Table S3) and males (Table 5). In contrast, in females, all vitamins, except for vitamin A1, the beta-carotene equivalent, vitamin D, and vitamin B3, showed a negative correlation with depressive symptoms. Deficiencies in vitamin group B showed the strongest correlation with an increase in the prevalence of depressive symptoms (Table 5).

We clarified the relationship between vitamin intake levels and depressive symptoms in females with different BMIs. In an analysis of overweight participants, a correlation was observed between vitamin intake and depressive symptoms for the retinol equivalent, vitamin D, vitamin K, vitamin B2, vitamin B5, and vitamin B9. No correlations were noted between vitamin intake and depressive symptoms in underweight females (Table 6). Furthermore, we did not find any significant association between depressive symptoms and vitamin intake in normal weight and overweight males (Supplementary Table S4)

## 4. Discussion

The results of the present cross-sectional study suggested that among elderly Japanese individuals, vitamin intake was lower in participants with depressive symptoms than in participants without depressive symptoms, particularly in females and those who were overweight. Moreover, vitamin intake was negatively associated with depressive symptoms in these groups. To the best of our knowledge, this is the first study to demonstrate an inverse relationship between vitamin intake and depressive symptoms in elderly Japanese female and overweight participants.

The present results confirmed previous findings showing that vitamin intake is inversely associated with the diagnosis of depression. Vitamin group B plays a major role in health, with deficiencies being linked to symptoms of psychiatric disorders and depression as well as altered memory function, cognitive impairment, and dementia [16,43]. In the present study, participants with depressive symptoms had lower levels of all vitamin group B, and a logistic regression with adjustments for confounders confirmed the negative relationship between depressive symptoms and the levels of vitamin B1, B5, B6, and B9 in all participants. Beside vitamin group B, the effects of other vitamins related to depression have been revealed. The effects of vitamin K on the central nervous system have mainly been studied under in vitro conditions. Based on its antioxidant actions [44], vitamin K has been shown to protect neurons and oligodendrocytes from oxidative damage [45] and the naphthoquinone ring was found to be responsible for this neuroprotective action [46]. Our results revealed a negative relationship between vitamin K and depressive symptoms, which is consistent with the findings of an experimental study on rats [47]. However, a study using Center for Epidemiologic Studies Depression Scale (CES-DS) for Children on school children aged 6–9 years old in Valencia, Spain, identified a positive relationship between children with depressive symptoms and vitamin K [48]. The difference observed between these findings may be due to the difference in target subjects (children vs elderly population) and depression scale (CES-DS vs. GDS). On the other hand, findings on the effects of vitamin C on depression in healthy individuals and patients with various medical conditions are contradictory. Randomized double-blind, placebo-controlled trial studies showed that vitamin C elevated the mood [49] and decreased the severity of depression [50]. Nevertheless, supplementary vitamin C did not decrease the depression score in type 2 diabetic patients [51]. Our results suggest that a deficiency in vitamin C increases the prevalence of depressive symptoms. Furthermore, the results showing a relationship between a decrease in cryptoxanthine levels and depression were consistent with previous findings [52].

Among female subjects, a relationship between depressive symptoms and vitamin intake was observed for six out of the seven vitamins in vitamin group B (except for vitamin B3), vitamin C, vitamin K, the retinol equivalent, alpha-tocopherol, and cryptoxanthine. Among male participants, although vitamin K intake was significantly lower in the participants with depressive symptoms than in participants without depressive symptoms, by regression analysis, no association was observed between vitamin intake and depressive symptoms. The present results are consistent with the findings of previous studies that investigated the relationship between vitamin group B and the prevalence of depression [27,53,54]. Previous findings indicated that a higher dietary intake of vitamin B6 among women and vitamin B12 among men is protective against late-life depression among generally healthy seniors living in the community [53]. A cross-sectional study among men aged between 40 and 60 years old in Finland did not find any relationships between cobalamin, pyridoxine, riboflavin, and depressive symptoms, but did for folate after adjustments [55]. In the present study, no significant differences were observed in vitamin D intake between groups with or without depressive symptoms, which was in contrast to previous findings [21,56]. The relationship between serum 25(OH)D concentrations and the prevalence of depression has been suggested to be stronger among women than men. An integrative review has suggested a significant association between mood disorders and low vitamin D levels, indicating that some biochemical mechanism may exist between the two variables [57]. In addition, a study on older adult males in Europe revealed an inverse relationship between 25(OH)D levels and depression that was largely independent of several lifestyle and health factors. Nevertheless, our results were similar to those obtained in studies conducted in China and the US [58,59]. Moreover, even though vitamin D has been suggested to protect against depression, this was not evident among older adults [60]. However, most of these studies focused on general subjects, limiting appropriate comparisons with our results to different genders.

Since depression and being overweight/obese are now very common, they have become popular areas of study. Although the relationship between obesity and depression has been examined, their causal relationship has not yet been elucidated. The present study focused on the relationship between vitamin intake and depressive symptoms in different BMI groups and the results obtained suggested that depressive symptoms were negatively associated with vitamin intake in the normal weight group and overweight group, but not in the underweight group. The present results showed a negative correlation between the retinol equivalent, vitamin K, vitamin B6, vitamin B9, and vitamin C and depressive symptoms in higher BMI groups. BMI is considered to be an important biomarker of malnutrition [61], and is closely related to depression in older adults [62]. Moreover, being overweight/obese has been proven to correlate with depression [63,64]. One of the reasons for this may be a diet imbalance. The diet consumed by Japanese individuals is typically characterized by a high intake of rice, soya products, fish, seaweed, and green tea and a low intake of animal fat and soft drinks [65]. In cross-sectional studies on the general population, a healthy Japanese dietary pattern, characterized by a higher intake of vegetables, fruit, mushrooms, soya products, vitamin B9, and vitamin C, was associated with a lower prevalence of depressive symptoms [66,67]. The present results showed a higher carbohydrate intake in males and females with depression than in non-depressed participants; therefore, we cannot completely eliminate the risk associated with high carbohydrate foods, which may lead to obesity, with low vitamin levels. Moreover, although consistent misreporting across all types of foods may not have influenced dietary energy density estimates, previous studies indicated that overweight individuals may selectively under-report their intake of fatty or sugary foods, which may result in the underestimation of dietary energy density as well as other nutrients. 

### Limitations

Several limitations of the present study warrant mention. The cross-sectional design of the present study does not permit the assessment of causality owing to the uncertain temporality of the association. All self-reported dietary assessment methods are subject to random and systematic measurement errors and the misreporting of dietary intakes, particularly by overweight/obese individuals, is also a limitation associated with self-reported dietary assessment methods [68]. Besides that, using an estimated questionnaire to determine nutrient intake may not give accurate results in terms of the value of nutrients taken from blood serum. Therefore, the nutrient intake values in this study may not be an exact representative value for the whole population. In addition, although we adjusted for various potential confounding variables, residual confounding variables such as physical activity, economic income, a history of drug use (particularly for participants with chronic diseases), a history of related diseases such as dementia (diagnosed by physicians), and a history of using vitamin supplements cannot be ruled out. 

## 5. Conclusions

The results of the present observational study indicate a relationship between depressive symptoms and vitamin deficiencies, particularly in female and overweight elderly participants. However, these participants may have had reduced dietary intakes of B vitamins, vitamin K, vitamin C, and others due to an imbalanced daily diet. Further studies using serum vitamins in blood in different gender and BMI groups need to be conducted in order to confirm the results of the present study.

## Figures and Tables

**Table 1:** Participant characteristics.

		Male			Female		
		Non-Depressive Symptoms (*n* = 528)	Depressive Symptoms (*n* = 192)	*p*1	Non-Depressive Symptoms (*n* = 669)	Depressive Symptoms (*n* = 245)	*p*2
Age (years) (mean ± SD)		73.0 ± 6.6	75.1 ± 7.3	0.001	74.10 ± 7.3	78.3 ± 8.2	<0.001
Height (cm) (mean ± SD)		163.6 ± 6.1	162.8 ± 6.4	0.091	150.1 ± 5.7	148.1 ± 6.6	<0.001
Weight (kg) (mean ± SD)		62.5 ± 9.3	61.6 ± 10.0	0.224	51.6 ±8.2	47.9 ± 8.1	<0.001
BMI (mean ± SD)		23.3 ± 2.9	23.3 ± 3.3	0.707	22.9 ± 3.2	21.8 ± 3.4	<0.001
BMI groups	Underweight (115)	22 (4.2%)	10 (5.2%)	0.741	46 (6.9%)	37 (15.1%)	<0.001
	Normal weight (1141)	371 (70.3%)	130 (67.7%)		466 (69.7%)	174 (71.0%)	
	Overweight (378)	135 (35.6%)	52 (27.1%)		157 (23.5%)	34 (13.9%)	
Energy	(kcal/day)	2058.7 ± 644.4	1893.0 ± 582.1	0.001	1697.7 ± 544.3	1607.3 ± 557.0	0.027
Protein	(g/1000 kcal)	38.1 ± 7.8	38.3 ± 8.1	0.744	40.9 ± 8.6	38.8 ± 7.8	0.001
Lipids (g/1000 kcal)		26.1 ± 6.2	26.2 ± 6.4	0.846	28.9 ± 6.5	27.5 ± 6.8	0.005
Carbohydrates (g/1000 kcal)		133.9 ± 22.2	137.8 ± 21.8	0.036	139.9 ± 20.3	144.7 ± 19.8	0.001

BMI: body mass index; SD: standard deviation.

**Table 2:** Differences in vitamin intake between the groups with and without depressive symptoms.

Vitamin (g/1000 kcal)	Non-Depressive Symptoms (*n* = 1197)	Depressive Symptoms (*n* = 437)	*p*
	Vitamin Intake (mean ± SD)		
Retinol	218.11 ± 266.03	197.54 ± 169.56	0.132
Retinol equivalent	401.32 ± 294.33	361.63 ± 203.53	0.011
Beta-carotene equivalent	2176.61 ± 1412.54	1958.11 ± 1368.00	0.005
Vitamin D	9.64 ± 5.75	9.05 ± 5.53	0.064
Alpha-tocopherol	4.02 ± 1.15	3.08 ± 1.11	0.001
Vitamin K	181.96 ± 100.73	156.02 ± 89.51	<0.001
Vitamin B1	0.42 ± 0.10	0.41 ± 0.10	0.002
Vitamin B2	0.72 ± 0.21	0.69 ± 0.20	0.007
Vitamin B3	9.48 ± 2.89	8.80 ± 2.64	<0.001
Vitamin B5	3.58 ± 0.79	3.45 ± 0.72	0.002
Vitamin B6	0.73 ± 0.20	0.68 ± 0.18	<0.001
Vitamin B9	190.29 ± 75.91	171.72 ± 65.14	<0.001
Vitamin B12	6.35 ± 3.35	5.84 ± 3.09	0.006
Vitamin C	65.78 ± 32.96	58.61 ± 30.65	<0.001
Cryptoxanthine	165.19 ± 148.49	145.59 ± 152.49	0.019

SD: standard deviation.

**Table 3:** Differences in vitamin intake between the groups with and without depressive symptoms by gender.

Vitamin (g/1000 kcal)	Male (*n* = 720)			Female (*n* = 914)		
	Non-Depressive Symptoms (*n* = 528, 73.3%)	Depressive Symptoms (*n* = 192, 26.7%)	*p*1	Non-Depressive Symptoms (*n* = 669, 73.2%)	Depressive Symptoms (*n* = 245, 26.8%)	*p*2
	Vitamin Intake (mean ± SD)			Vitamin Intake (mean ± SD)		
Retinol	237.11 ± 361.04	223.85 ± 221.95	0.633	203.11 ± 152.85	176.92 ± 108.81	0.004
Retinol equivalent	394.48 ± 377.24	368.73 ± 242.38	0.378	406.72 ± 206.79	357.85 ± 167.28	<0.001
Beta-carotene equivalent	1866.66 ± 1232.40	1715.04 ± 1181.41	0.140	2421.24 ± 1496.10	2148.59 ± 1472.59	0.014
Vitamin D	9.10 ± 5.37	9.35 ± 5.63	0.590	10.06 ± 6.01	8.82 ± 5.46	0.005
Alpha-tocopherol	3.69 ± 1.04	3.65 ± 1.04	0.609	4.28 ± 1.17	3.93 ± 1.16	<0.001
Vitamin K	165.52 ± 89.82	148.69 ± 83.04	0.024	194.94 ± 106.87	161.76 ± 94.01	<0.001
Vitamin B1	0.39 ± 0.09	0.39 ± 0.09	0.578	0.45 ± 0.10	0.42 ± 0.10	0.001
Vitamin B2	0.68 ± 0.20	0.68 ± 0.20	0.969	0.74 ± 0.21	0.69 ± 0.20	<0.001
Vitamin B3	9.20 ± 2.78	8.82 ± 2.53	0.095	9.70 ± 2.95	8.77 ± 2.73	<0.001
Vitamin B5	3.39 ± 0.73	3.39 ± 0.72	0.933	3.74 ± 0.81	3.50 ± 0.72	<0.001
Vitamin B6	0.70 ± 0.18	0.68 ± 0.18	0.155	0.75 ± 0.21	0.69 ± 0.19	<0.001
Vitamin B9	178.17 ± 70.28	168.11 ± 62.62	0.081	199.87 ± 78.83	174.56 ± 67.03	<0.001
Vitamin B12	6.22 ± 3.32	6.12 ± 3.31	0.708	6.45 ± 3.37	5.62 ± 2.91	0.001
Vitamin C	57.45 ± 29.89	55.11 ± 32.27	0.363	72.34 ± 33.79	61.36 ± 29.09	<0.001
Cryptoxanthine	132.53 ± 124.92	142.65 ± 176.63	0.394	190.97 ± 160.18	147.89 ± 130.84	<0.001

SD: standard deviation.

**Table 4:** Differences in vitamin intake between the groups with and without depressive symptoms by BMI.

Vitamin (g/1000 kcal)	Underweight			Normal Weight			Overweight		
	Non-Depressive Symptoms (*n* = 68)	Depressive Symptoms (*n* = 47)	*p*1	Non-Depressive Symptoms (*n* = 837)	Depressive Symptoms (*n* = 304)	*p*2	Non-Depressive Symptoms (*n* = 292)	Depressive Symptoms (*n* = 86)	*p*3
	Vitamin Intake (mean ± SD)			Vitamin Intake (mean ± SD)			Vitamin Intake (mean ± SD)		
Retinol	210.08 ± 151.47	193.65 ± 156.16	0.573	218.76 ± 294.58	204.49 ± 183.80	0.429	218.09 ± 190.09	175.06 ± 114.67	0.010
Retinol equivalent	388.40 ± 194.04	386.42 ± 199.02	0.958	405.21 ± 318.96	374.21 ± 215.49	0.117	393.19 ± 234.44	308.70 ± 147.56	<0.001
Beta-carotene equivalent	2117.37 ± 1544.73	2286.89 ± 1619.20	0.572	2215.12 ± 1395.75	2013.97 ± 1405.64	0.033	2080.02 ± 1428.29	1580.93 ± 960.37	<0.001
Vitamin D	9.29 ± 5.09	10.14 ± 5.54	0.398	9.66 ± 5.88	9.10 ± 5.46	0.150	9.67 ± 5.55	8.27 ± 5.75	0.043
Alpha-tocopherol	4.05 ± 1.09	4.02 ± 1.28	0.897	4.02 ± 1.17	3.83 ± 1.09	0.016	4.02 ± 1.11	3.58 ± 1.08	0.001
Vitamin K	180.55 ± 98.0	153.55 ± 86.23	0.130	183.08 ± 100.47	162.02 ± 92.38	0.001	179.10 ± 102.37	136.16 ± 78.27	<0.001
Vitamin B1	0.42 ± 0.10	0.43 ± 0.11	0.461	0.42 ± 0.10	0.41 ± 0.09	0.037	042 ± 0.10	0.38 ± 0.09	0.001
Vitamin B2	0.72 ± 0.20	0.70 ± 0.18	0.600	0.72 ± 0.21	0.69 ± 0.21	0.145	0.72 ± 0.22	0.65 ± 0.19	0.005
Vitamin B3	9.17 ± 2.63	8.81 ± 2.79	0.488	9.49 ± 2.91	8.90 ± 2.55	0.001	9.55 ± 2.89	8.41 ± 2.88	0.001
Vitamin B5	3.62 ± 0.76	3.56 ± 0.66	0.707	3.58 ± 0.79	3.48 ± 0.72	0.043	3.59 ± 0.81	3.31 ± .073	0.003
Vitamin B6	0.72 ± 0.20	0.71 ± 0.20	0.829	0.73 ± 0.20	0.69 ± 0.18	0.002	0.73 ± 0.19	0.64 ± 0.19	<0.001
Vitamin B9	185.01 ± 69.89	174.95 ± 69.57	0.449	191.74 ± 76.54	176.09 ± 66.01	0.001	187.39 ± 75.58	154.52 ± 56.90	<0.001
Vitamin B12	6.10 ± 3.01	6.08 ± 3.37	0.966	6.33 ± 3.39	5.92 ± 3.06	0.065	6.48 ± 3.33	5.44 ± 3.09	0.010
Vitamin C	63.48 ± 29.69	60.49 ± 35.16	0.623	66.30 ± 33.55	60.32 ± 3.17	0.005	64.81 ± 32.01	51.51 ± 24.98	<0.001
Cryptoxanthine	158.82 ± 124.14	148.58 ± 162.53	0.703	164.99 ± 149.82	146.52 ± 153.02	0.067	167.25 ± 150.27	140.65 ± 146.56	0.148

SD: standard deviation.

**Table 5:** Odds ratios (95% CI) for depressive symptom outcomes according to vitamin intake stratified by gender *.

Vitamin (g/1000 kcal)	Male			Female		
	B	OR (95% CI)	*p*	B	OR (95% CI)	*p*
Retinol	0.000	1.000 (0.999–1.000)	0.779	−0.001	0.999 (0.997–1.000)	0.098
Retinol equivalent	0.000	1.000 (0.999–1.000)	0.459	−0.001	0.999 (0.997–1.000)	0.006
Beta-carotene equivalent	0.000	1.000 (1.000–1.000)	0.065	0.000	1.000 (1.000–1.000)	0.024
Vitamin D	0.037	1.038 (0.998–1.080)	0.063	−0.032	0.969 (0.935–1.004)	0.080
Alpha-tocopherol	0.014	1.014 (0.830–1.239)	0.889	−0.239	0.788 (0.645–0.962)	0.020
Vitamin K	−0.002	0.998 (0.996–1.000)	0.052	−0.003	0.997 (0.995–0.999)	0.001
Vitamin B1	−0.585	0.557 (0.066–4.729)	0.592	−2.148	0.117 (0.014–0.949)	0.044
Vitamin B2	−0.045	0.956 (0.345–2.649)	0.931	−1.183	0.306 (0.102–0.921)	0.035
Vitamin B3	−0.019	0.981 (0.902–1.067)	0.652	−0.082	0.921 (0.847–1.002)	0.055
Vitamin B5	−0.013	0.987 (0.754–1.291)	0.923	−0.388	0.678 (0.511–0.901)	0.007
Vitamin B6	−0.323	0.724 (0.213–2.458)	0.605	−1.656	0.191 (0.064–0.570)	0.003
Vitamin B9	−0.003	0.997 (0.995–1.000)	0.073	−0.005	0.995 (0.993–0.998)	0.000
Vitamin B12	0.029	1.030 (0.964–1.099)	0.383	−0.079	0.924 (0.862–0.989)	0.023
Vitamin C	−0.004	0.996 (0.990–1.003)	0.245	−0.010	0.990 (0.985–0.996)	0.001
Cryptoxanthine	0.000	1.000 (0.999–1.002)	0.542	−0.002	0.998 (0.997–0.999)	0.003

* Adjusted for Age, Height, Weight, BMI, Living status, Marital status, Drinking alcohol, Smoking status, Energy, Carbohydrates, Hypertension, Diabetes, and Hyperlipidemia. B: beta coefficient; OR: Odd ratio, CI: confidence interval.

**Table 6:** Odds ratios (95% CI) for depressive symptom outcomes according to vitamin intake stratified by BMI in females *.

Vitamin (g/1000 kcal)	B	Underweight	*p*	B	Normal Weight	*p*	B	Overweight	*p*
		OR (95% CI)			OR (95% CI)			OR (95% CI)	
Retinol	0.000	1.000 (0.994–1.007)	0.892	−0.002	0.998 (0.997–1.000)	0.134	−0.005	0.995 (0.990–1.000)	0.061
Retinol equivalent	0.000	1.000 (0.996–1.004)	0.991	−0.002	0.998 (0.997–1.000)	0.012	−0.004	0.996 (0.992–0.999)	0.022
Beta-carotene equivalent	0.000	1.000 (1.000–1.000)	0.926	0.000	1.000 (1.000–1.000)	0.039	0.000	1.000 (0.999–1.000)	0.201
Vitamin D	0.080	1.083 (0.934–1.257)	0.290	−0.029	0.971 (0.933–1.011)	0.153	−0.133	0.876 (0.771–0.995)	0.042
Alpha-tocopherol	−0.299	0.742 (0.369–1.492)	0.402	−0.253	0.777 (0.613–0.983)	0.036	−0.185	0.831 (0.449–1.538)	0.555
Vitamin K	−0.007	0.993 (0.985–1.000)	0.056	−0.002	0.998 (0.996–1.000)	0.043	−0.009	0.992 (0.985–0.998)	0.015
Vitamin B1	−1.998	0.136 (0.00–229.57)	0.598	−2.552	0.078 (0.006–0.963)	0.047	−4.167	0.016 (0.000–6.160)	0.172
Vitamin B2	−1.668	0.189 (0.01–30.431)	0.520	−0.850	0.427 (0.119–1.535)	0.192	−3.852	0.021 (0.001–0.479)	0.015
Vitamin B3	0.095	1.099 (0.804–1.503)	0.553	−0.082	0.921 (0.834–1.017)	0.104	−0.136	0.873 (0.692–1.102)	0.254
Vitamin B5	−0.648	0.523 (0.169–1.619)	0.261	−0.309	0.734 (0.524–1.029)	0.072	−0.950	0.387 (0.181–0.826)	0.014
Vitamin B6	−1.056	0.348 (0.008–15.34)	0.585	−1.737	0.176 (0.047–0.657)	0.010	−2.692	0.068 (0.003–1.433)	0.084
Vitamin B9	−0.004	0.996 (0.985–1.006)	0.419	−0.004	0.996 (0.993–0.999)	0.005	−0.010	0.990 (0.982–0.999)	0.023
Vitamin B12	0.108	1.114 (0.842–1.473)	0.450	−0.077	0.926 (0.855–1.003)	0.060	−0.196	0.822 (0.672–1.006)	0.057
Vitamin C	−0.008	0.992 (0.972–1.012)	0.426	−0.010	0.991 (0.984–0.997)	0.004	−0.014	0.986 (0.969–1.003)	0.112
Cryptoxanthin	−0.002	0.998 (0.994–1.002)	0.361	−0.002	0.998 (0.996–0.999)	0.007	−0.001	0.999 (0.996–1.002)	0.588

* Adjusted for Age, Height, Weight, BMI, Living status, Marital status, Drinking alcohol, Smoking status, Energy, Carbohydrates, Hypertension, Diabetes, and Hyperlipidemia. B: beta coefficient, OR: odd ratio, CI: confidence interval.

## Supplementary Materials

The following are available online at www.mdpi.com/2072-6643/9/12/1319/s1, Table S1: Participant characteristics stratified by depressive symptoms and non-depressive symptoms groups. Table S2: Odds ratios (95% CI) for depressive symptom outcomes according to vitamin intake in all participants. Table S3: Odds ratios (95% CI) for depressive symptom outcomes according to vitamin intake stratified by BMI in all participants. Table S4: Odds ratios (95% CI) for depressive symptom outcomes according to vitamin intake stratified by BMI in males.

## Abbreviations

BDHQ	Brief-type self-administered diet history questionnaire
GDS	Geriatric Depression Scale
BMI	Body mass index
25(OH)D	25-hydroxyvitamin D
SPSS	Statistical Package for Social Sciences
SD	Standard deviation
B	Beta coefficient
OR	Odd ratio
CI	Confidence interval
