# Supplementary material for: Relationship between Vitamin Intake and Depressive Symptoms in Elderly Japanese Individuals: Differences with Gender and Body Mass Index
Source: Nutrients. 2017 Dec 3;9(12):1319. doi: 10.3390/nu9121319 (PMC5748769; doi:10.3390/nu9121319)
Supplement: Supplementary file 1 [file nutrients-09-01319-s001.pdf]

Relationship between vitamin intake and depressive symptoms in elderly Japanese individuals: Differences with gender and body mass index

Supplementary

Table S1: Participant characteristics stratified by depressive symptoms and non-depressive symptoms groups

		Non-depressive symptoms (n=1197)	Depressive symptoms (n=437)	p
Gender	Male (n=720) (n,%)	528 (73.33)	192 (26.67)	0.950 **
	Female (n=914) (n,%)	669 (73.19)	245 (26.81)	
Age (mean ± SD)		73.59 ± 7.02	76.89 ± 7.97	<0.001*
Height (mean ± SD)		156.04 ± 8.93	154.55 ± 9.76	0.005*
Body weight (mean ± SD)		56.39 ± 10.24	53.90 ± 11.23	<0.001*
BMI (mean ± SD)		23.06 ± 3.06	22.43 ± 3.41	<0.001*
	Underweight (n=115) (n,%)	68 (59.13)	47 (40.87)	
BMI group	Normal weight (n=1141) (n,%)	837 (73.36)	304 (26.64)	0.001**
	Overweight	292 (77.25)	86 (22.75)	
Energy kcal/day (mean ± SD)		1856.96 ± 616.97	1732.83 ± 585.03	<0.001*
Protein (mean ± SD)		39.63 ± 3.27	38.55 ± 7.91	0.020*
Lipid (mean ± SD)		27.70 ± 6.51	26.97 ± 6.61	0.047*
Carbohydrate (mean ± SD)		137.26 ± 21.36	141.68 ± 20.96	<0.001*

** Chi-square test; * t-test; SD: standard deviation

Table S2: Odds ratios (95% CI) for depressive symptoms outcomes according to vitamin intake in all participants

Vitamin (g/1000 kcal)	Univariate			Multivariate*		
	B	OR (95%CI)	p	B	OR (95%CI)	p
Vitamin A1	-0.001	0.999(0.999-1.000)	0.111	0.000	1.000 (0.999-1.000)	0.364
Beta-carotene equivalent	0.000	1.000 (1.000-1.000)	0.006	0.000	1.000 (1.000-1.000)	0.001
Retinol equivalent	-0.001	0.999 (0.999-1.000)	0.005	-0.001	0.999 (0.999-1.000)	0.017
Vitamin D	-0.019	0.981 (0.962-1.001)	0.065	-0.002	0.998 (0.973-1.024)	0.876
Alpha-tocopherol	-0.169	0.844 (0.765-0.932)	0.001	-0.149	0.861 (0.753-0.985)	0.030

Vitamin K	-0.003	0.997 (0.996-0.998)	<0.001	-0.003	0.997 (0.996-0.999)	<0.001
Vitamin B1	-1.684	0.186 (0.061-0.563)	0.003	-1.495	0.224 (0.054-0.927)	0.039
Vitamin B2	-0.748	0.473 (0.274-0.819)	0.007	-0.608	0.544 (0.265-1.117)	0.097
Vitamin B3	-0.089	0.915 (0.879-0.953)	<0.001	-0.048	0.953 (0.900-1.009)	0.098
Vitamin B5	-0.224	0.800 (0.692-0.924)	0.003	-0.201	0.818 (0.679-0.984)	0.033
Vitamin B6	-1.217	0.296 (0.165-0.531)	<0.001	-1.099	0.333 (0.152-0.730)	0.006
Vitamin B9	-0.004	0.996 (0.995-0.998)	<0.001	-0.004	0.996 (0.994-0.998)	<0.001
Vitamin B12	-0.050	0.952 (0.919-0.986)	0.006	-0.025	0.975 (0.931-1.021)	0.289
Vitamin C	-0.007	0.993 (0.989-0.996)	<0.001	-0.008	0.992 (0.988-0.996)	<0.001
Cryptoxanthine	-0.001	0.999 (0.998-1.000)	0.020	-0.001	0.999 (0.998-1.000)	0.036

*Adjusted for Gender, Age, Height, Weight, BMI, Living condition, Marital status, Drinking alcohol, Smoking status, Energy, Carbohydrates, Hypertension, Diabetes, Hyperlipidemia
B: beta coefficient; OR: odd ratio, CI: confidence interval

Table S3: Odds ratios (95% CI) for depressive symptoms outcomes according to vitamin intake stratified by BMI in all participants

Vitamin	Underweight			Normal weight			Overweight		
(g/1000 kcal)	B	OR (95%CI)	p	B	OR (95%CI)	p	B	OR (95%CI)	p
Vitamin A1	-0.001	0.999 (0.995-1.003)	0.648	0.000	1.000 (0.999-1.000)	0.688	-0.001	0.999 (0.997-1.001)	0.253
Beta-carotene equivalent	0.000	1.000 (1.000-1.000)	0.950	0.000	1.000 (1.000-1.000)	0.006	0.000	1.000 (1.000-1.000)	0.046
Retinol equivalent	0.000	1.000 (0.997-1.003)	0.795	-0.001	0.999 (0.999-1.000)	0.155	-0.002	0.998 (0.996-1.000)	0.040
Vitamin D	0.104	1.110 (0.984-1.251)	0.089	-0.007	0.993 (0.964-1.024)	0.669	0.001	1.001 (0.943-1.063)	0.975
Alpha-tocopherol	-0.036	0.965 (0.574-1.620)	0.892	-0.146	0.864 (0.735-1.015)	0.075	-0.139	0.870 (0.640-1.183)	0.375
Vitamin K	-0.002	0.998 (0.992-1.003)	0.396	-0.002	0.998 (0.996-0.999)	0.003	-0.004	0.996 (0.992-0.999)	0.014
Vitamin B1	0.027	1.027 (0.003-308.64)	0.993	-1.452	0.234 (0.042-1.309)	0.098	-2.994	0.050 (0.002-1.248)	0.068
Vitamin B2	0.085	1.089 (0.027-43.299)	0.964	-0.466	0.628 (0.269-1.467)	0.282	-1.188	0.305 (0.061-1.530)	0.149
Vitamin B3	0.055	1.056 (0.816-1.367)	0.678	-0.042	0.959 (0.896-1.026)	0.223	-0.056	0.946 (0.832-1.075)	0.392
Vitamin B6	-0.402	0.669 (0.040-11.171)	0.780	-0.920	0.398 (0.155-1.023)	0.056	-1.986	0.137 (0.023-0.832)	0.031
Vitamin B12	0.093	1.097 (0.882-1.365)	0.407	-0.021	0.979 (0.927-1.034)	0.452	-0.036	0.965 (0.865-1.076)	0.519
Vitamin B9	-0.001	0.999 (0.991-1.006)	0.723	-0.004	0.996 (0.994-0.999)	0.002	-0.006	0.994 (0.990-0.999)	0.013
Vitamin B5	-0.118	0.888 (0.393-2.007)	0.776	-0.185	0.831 (0.665-1.038)	0.103	-0.288	0.750 (0.497-1.129)	0.168
Vitamin C	-0.004	0.997 (0.982-1.012)	0.650	-0.006	0.994 (0.989-0.998)	0.009	-0.013	0.987 (0.978-0.997)	0.014
Cryptoxanthine	-0.001	0.999 (0.996-1.002)	0.468	-0.001	0.999 (0.998-1.000)	0.096	-0.001	0.999 (0.998-1.001)	0.613

*Adjusted for Age, Height, Weight, BMI, Living condition, Marital status, Drinking alcohol, Smoking status, Energy, Carbohydrates, Hypertension, Diabetes, Hyperlipidemia
B: beta coefficient; OR: odd ratio, CI: confidence interval

Table S4: Odds ratios (95% CI) for depressive symptoms outcomes according to vitamin intake stratified by BMI in males

Vitamin (g/1000	Normal weight			Over weight		
kcal)	B	OR (95%CI)	P	B	OR (95%CI)	P

Vitamin A1	0.000	1.000 (0.999-1.001)	0.987	-0.001	0.999 (0.997-1.002)	0.550
Beta-carotene equivalent	0.000	1.000 (1.000-1.000)	0.104	0.000	1.000 (0.999-1.000)	0.242
Retinol equivalent	0.000	1.000 (0.999-1.000)	0.733	-0.001	0.999 (0.996-1.001)	0.279
Vitamin D	0.029	1.029 (0.979-1.082)	0.265	0.048	1.049 (0.972-1.132)	0.217
Alpha-tocopherol	0.015	1.015 (0.797-1.291)	0.905	-0.070	0.932 (0.605-1.437)	0.751
Vitamin K	-0.003	0.997 (0.995-1.000)	0.045	-0.002	0.998 (0.993-1.003)	0.430
Vitamin B1	0.066	1.068 (0.079-14.401)	0.961	-3.304	0.037 (0.000-3.619)	0.158
Vitamin B2	0.021	1.021 (0.310-3.366)	0.973	-0.386	0.680 (0.066-6.977)	0.745
Vitamin B3	-0.010	0.990 (0.896-1.094)	0.847	-0.064	0.938 (0.781-1.127)	0.496
Vitamin B6	0.193	1.213 (0.274-5.369)	0.799	-1.973	0.139 (0.010-2.008)	0.148
Vitamin B12	0.033	1.033 (0.955-1.118)	0.415	0.032	1.032 (0.893-1.193)	0.667
Vitamin B9	-0.002	0.998 (0.994-1.001)	0.190	-0.004	0.996 (0.989-1.002)	0.204
Vitamin B5	-0.045	0.956 (0.692-1.320)	0.785	-0.027	0.973 (0.543-1.746)	0.928
Vitamin C	-0.001	0.999 (0.991-1.006)	0.746	-0.013	0.987 (0.973-1.002)	0.095
Cryptoxanthine	0.001	1.001 (0.999-1.002)	0.401	0.000	1.000 (0.997-1.003)	0.934

*Adjusted for Gender, Age, Height, Weight, BMI, Living condition, Marital status, Drinking alcohol, Smoking status, Energy, Carbohydrates, Hypertension, Diabetes, Hyperlipidemia
B: beta coefficient; OR: odd ratio, CI: confidence interval
